# Differences in the incidence of cirrhosis-associated complications between MASLD, MetALD and ALD among patients with decompensated liver cirrhosis

**DOI:** 10.1371/journal.pone.0325673

**Published:** 2025-06-26

**Authors:** Laura Buttler, Anja Tiede, Marie Griemsmann, Hannah Rieland, Jim B. Mauz, Heiner Wedemeyer, Markus Cornberg, Tammo L. Tergast, Katharina L. Hupa-Breier, Benjamin Maasoumy

**Affiliations:** 1 Department of Gastroenterology, Hepatology, Infectious Diseases and Endocrinology, Hannover Medical School, Hannover, Germany; 2 German Centre for Infection Research (DZIF), partner-site Hannover-Braunschweig, Hannover, Germany; 3 RESIST Cluster of Excellence, Hannover Medical School, Hannover, Germany; 4 Centre for Individualised Infection Medicine (CiiM), A Joint Venture Between the Helmholtz Centre for Infection Research (HZI) and Hannover Medical School (MHH), Hannover, Germany; 5 TWINCORE, A Joint Venture Between the Helmholtz-Centre for Infection Research (HZI) and the Hannover Medical School (MHH), Hannover, Germany; Tan Tao University, VIET NAM

## Abstract

**Background:**

Recently, the new definition of steatotic liver disease (SLD) has been introduced, which not only differentiates MASLD (Metabolic Dysfunction-Associated steatotic liver disease) from alcohol-related steatotic liver disease (ALD), but also introduces the concept of metabolic and alcohol-related SLD (MetALD). However, potential differences of the new etiologies regarding the clinical phenotype of patients with advanced liver cirrhosis still remain undetermined. Therefore, we analyzed survival and the incidence of cirrhosis-related complications in SLD-patients with advanced liver cirrhosis.

**Methods:**

A number of 416 consecutive patients with MASLD, MetALD- and ALD-associated decompensated liver cirrhosis were investigated. Overall survival, infections, hepatic encephalopathy, portal-hypertensive bleeding, rehospitalization and development of hepatocellular carcinoma were retrospectively analyzed within one year of follow-up. Cox regression analyses were performed for survival, competing risk analyses for the cirrhosis-specific complications. MASLD was used as reference group.

**Results:**

ALD was associated with a lower risk of infections (HR = 0.55; p < 0.001) compared to MASLD. This remained significant after adjustment for age, sex, Model for End-Stage Liver Disease (MELD), serum-sodium, serum-cholinesterase, diabetes, body mass index and norfloxacin (HR = 0.59; p = 0.02) in the multivariable competing risk model. Notably, the incidence of infections in MetALD patients was in between both groups (MetALD: 68.7%, ALD: 56.1%, MASLD: 87.3%). However, there were no differences in survival (MetALD: HR = 1.03; p = 0.93; ALD: HR = 0.79; p = 0.49) and the other complications studied here.

**Conclusion:**

The risk of infections is increased in MASLD-associated cirrhosis compared to other SLD-phenotypes. Thus, the role of a metabolic risk profile should not be neglected even in patients with decompensated liver cirrhosis.

## Introduction

Steatotic liver diseases (SLD) are highly prevalent in the general population and could be accompanied by the progression of hepatocyte damage, persistent inflammation, and increased fibrosis [1–4]. Consequently, SLD represents a main cause of liver cirrhosis [5]. Studies report that alcohol-related steatotic liver disease (ALD) is responsible for about two thirds of cirrhosis in Europe, but the number of end-stage liver disease caused by metabolic-associated liver damage increases rapidly [6]. Recently, a new concept of SLD has been implemented. This terminology does not longer differentiate non-alcoholic fatty liver disease (NAFLD) from alcohol-related liver disease, but introduces the subdivision in ALD, Metabolic Dysfunction-associated SLD (MASLD) and the novel MetALD group (metabolic and alcohol-related SLD), with the combination of alcohol-related and metabolic-dysfunction associated cirrhosis [7]. Patients of this new category are characterized by a weekly alcohol intake of 210–420 g (male) or 140–350 g (female) and fulfill at least one out of five cardiometabolic criteria. Therefore, this new concept acknowledges the co-incidence of cardiometabolic risk factors and additional alcohol-use [8].

If SLD, regardless its type, remains unaddressed and becomes chronic, patients are prone to progression of fibrosis, finally resulting in the irreversible state of liver cirrhosis [3]. Once decompensated, cirrhosis patients suffer from a range of complications, particularly including a high prevalence of infections [9]. Infections are present in about a third of hospitalized cirrhotic patients and are potential drivers for other types of decompensation, such as acute kidney injury, hydropic decompensation or hepatic encephalopathy (HE) [9]. Finally, decompensated cirrhosis in general, but especially with the occurrence of complications, is accompanied by a high mortality [10]. Hence, not only immediate treatment of complicating events, but also identification of risk factors is required to avert worsened outcomes of our patients. The need for risk stratification includes the question, whether the etiology of cirrhosis and etiology-associated comorbidities impact the prognosis by elevating the risk for certain complications.

However, the impact of the newly defined SLD etiologies on the clinical outcome of patients with decompensated liver disease has not been investigated, yet. Therefore, we aimed to compare overall survival and the incidence of the most relevant cirrhosis-associated complications between MASLD, MetALD and ALD-patients with decompensated liver cirrhosis.

## Methods

### Study cohort and design

A number of 416 consecutive patients with decompensated cirrhosis who underwent paracentesis at Hannover Medical School between 2011 and 2023 were investigated. Missing of written informed consent, age < 18 years, absence of underlying SLD and missing data about alcohol intake were defined as exclusion criteria. Diagnosis of NAFLD- or alcohol-related decompensated cirrhosis that was present in 544 subjects, was required for inclusion. A number of 128 subjects were excluded due to missing information on the extent of alcohol consumption, so that 416 patients remained for analyses. Cirrhosis etiology was reclassified according to the new SLD-definition as defined in “The multisociety Delphi consensus statement on new fatty liver disease nomenclature” [7]. ALD was defined as (former) alcohol intake of more than 420 g/week for male and 350 g/week for female patients. Those with alcohol intake of 210–420 g/week (male) or 140–350 g/week (female) who additionally fulfilled at least one out of five cardiometabolic criteria were considered as MetALD [7]. Etiology of patients with lower or without any alcohol consumption and the presence of at least one cardiometabolic criterium was defined as MASLD. Diagnosis of steatosis was based on imaging. The extent of alcohol consumption was extracted from the patients` medical reports. Patient files, medical and laboratory reports were accessed for collection of demographic and medical data.

The primary endpoint was survival. As secondary endpoints, the incidences of any bacterial infections, spontaneous bacterial peritonitis (SBP) and overt hepatic encephalopathy (oHE) were examined. Furthermore, portal-hypertensive bleeding and hepatic decompensation requiring rehospitalization were studied up to one year of follow-up. Occurrence of hepatocellular carcinoma (HCC), diagnosed by ultrasound, MRI or CT imaging, was investigated within five years. Here, a number of 21 patients with HCC at baseline were excluded, leaving 395 patients for analysis.

Diagnosis of oHE was based on the West Haven criteria [11], hepatic decompensation on the Baveno VII criteria [9]. Infections were defined as follows:

- SBP: At least 250 polymorphonuclear- or 500 nucleus-containing cells per microliter ascitic fluid (following paracentesis).
- Urinary tract infection (UTI): Significant leukocyturia and/or positive urine cultures with symptoms.
- Pneumonia: Infiltrates in X-ray and/or clinical symptoms.
- Blood stream infections: Positive blood cultures and clinical symptoms.
- Infection with unknown source: Signs of infections without identifiable source.

We performed cox regression analyses to compare overall survival. Patients were censored at time-point of liver transplantation (LTx) or end of follow-up. Competing risk analyses were used for the cirrhosis-associated complications. LTx and/or death were treated as competitors. In a second approach, we adjusted for potential confounders in a multivariable model. This model included the covariables age, sex, Model for End-Stage Liver Disease (MELD), sodium, diabetes mellitus, body mass index (BMI) and serum-cholinesterase (S-CHE), as this has been identified as important prognostic marker for patients with decompensated liver cirrhosis [12]. For analysis of infections and SBP, we additionally adjusted for norfloxacin intake. When investigating the incidences of oHE, intake of any HE-prophylaxis (rifaximin, lactulose and/or l-ornithine l-aspartate) was considered as additional covariable, whereas intake of non-selective betablockers was included in the multivariable model for portal-hypertensive bleeding. MASLD-etiology functioned as reference group in all analyses.

In a second approach, the cox regression analysis and the competing risk analyses were repeated after matching MASLD with ALD patients. Using nearest neighbor matching based on propensity score, a number of 48 MASLD patients were matched to ALD patients in a 1:1 ratio. The parameters age, platelet count, creatinine and diabetes were incorporated as matching variables. S1 and S2 Tables display the baseline parameters of both groups after matching and the results of the analyses, respectively. Given that MELD score and BMI still differed after matching, we adjusted for both in the multivariable models.

To demarcate the novel categorization in three SLD groups from the former division in two groups, we additionally combined the MetALD and ALD group and compared them with the MASLD patients. The results can be found in S3 Table.

Baseline was defined as time point of hospital admission. Information on every further visit at our outpatient clinic or on following rehospitalizations were used to generate follow-up data. Each analysis was performed in a short 90 days follow-up and in a long-term one year follow-up. Patients who did not complete the observational period were censored at time of loss to follow-up.

### Statistics

IBM SPSS Statistics (Version 28, IBM®, New York) and *R* Statistical Software (version 4.2.0, *R* foundation for statistical Computing, Vienna, Austria) with “tableone” package was used to analyze patients` characteristics. For Kaplan-Meier curves, cox regression and competing risk analyses, *R* Statistical Software (version 4.2.0, *R* foundation for statistical Computing, Vienna, Austria) with *R* commander and plugin ‘EZR’ was used.

Categorial variables (shown as number and percentage) were compared with a Chi-Square test. ANOVA was performed for continuous variables (presented as median and interquartile range). After matching, McNemar was utilized to compare categorial values, whereas Wilcoxon test was used for continuous variables. For matching, R Statistical Software with *R studio* and the *“MatchIt” package* was used.

### Ethics

Our study was performed in accordance with the ethics committee of Hannover Medical School (Nr. 7935_BO_K_2018) and respected the declarations of Helsinki. All patients gave written informed consent for data analyses.

## Results

### Cohort characterization and prevalence of SLD groups

**Fig 1:**
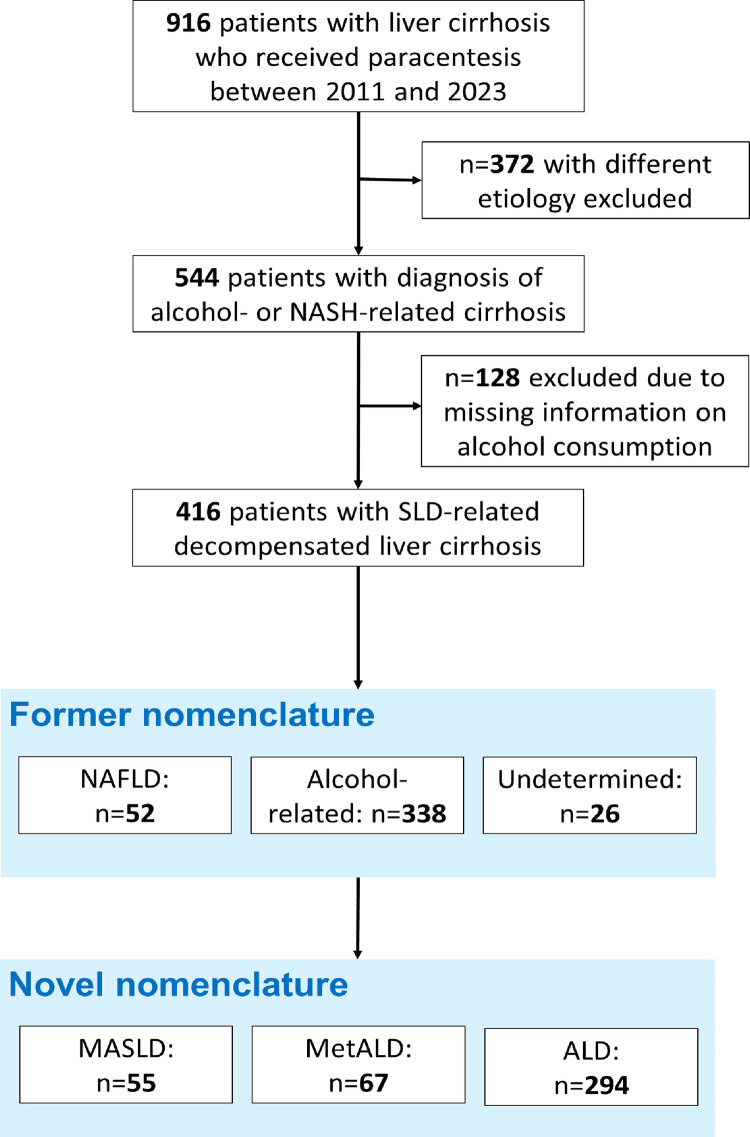
Study cohort. Figure 1 provides an overview of the included and excluded patients and the prevalence of SLD-types in the studied cohort. ALD: Alcohol-related steatotic liver disease; MASLD: Metabolic Dysfunction-Associated steatotic liver disease; MetALD: Metabolic and alcohol-related steatotic liver disease; NAFLD: Nonalcoholic fatty liver disease; NASH: Nonalcoholic steatohepatitis.

NAFLD- and alcohol-related decompensated liver cirrhosis was diagnosed in 12.5% (n = 52) and 81.3% (n = 338), respectively. Predominance of metabolic or alcohol-related etiology used to be undetermined in 6.3% (n = 26) of the patients. Implementing the new nomenclature, 13.2% (n = 55), 16.1% (n = 67) and 70.7% (n = 294) fulfilled the criteria for MASLD, MetALD and ALD, respectively (Fig 1). Median baseline MELD was 18 in MASLD and 17 in MetALD and ALD (p = 0.92), median age was 59 years in MASLD and MetALD patients and 56 years in the ALD group (p < 0.001). The prevalence of diabetes decreased stepwise from MASLD (n = 33; 60.0%) over MetALD (n = 23; 34.8%) to ALD (n = 45; 15,3%; p < 0.001). Likewise, the BMI reached highest values in MASLD patients, with a median BMI of 27.8 kg/$m^{2}$ and lowest values in the ALD group (22.1 kg/$m^{2}$; p < 0.001). Regarding the history of prior decompensating events, previous episodes of decompensation have been observed in over 80% of the patients, indicating their end-stage disease, but no differences were detected between groups (Table 1).

**Table 1:** Baseline characteristics. Baseline characteristics of the 416 analyzed patients with steatotic liver disease associated decompensated liver cirrhosis.

	All patients (n = 416)	MASLD (n = 55)	MetALD (n = 67)	ALD (n = 294)	p value^*^
**Sex**					0.288
** Male**	299 (71.9)	35 (63.6)	47 (70.1)	217 (73.8)	
** Female**	117 (28.1)	20 (36.4)	20 (29.9)	77 (26.2)	
**Age (years)**	56.8 (50.1-63.0)	59.2 (54.1-66.9)	59.0 (52.1-65.8)	55.6 (48.6-61.9)	<0.001
**SBP at baseline**	53 (12.7)	7 (12.7)	8 (11.9)	38 (12.9)	0.976
**Infection at baseline**	120 (28.8)	16 (29.1)	27 (40.3)	77 (26.2)	0.071
**Hepatic encephalopathy at baseline**	55 (13.2)	6 (10.9)	5 (7.5)	44 (15.0)	0.226
**Portal-hypertensive bleeding at baseline**	2 (0.5)	0 (0.0)	0 (0.0)	2 (0.7)	0.659
**MELD**	17 (13-23)	18 (14-22)	17 (14-23)	17 (13-24)	0.920
**Potassium (mmol/l)**	4.2 (3.7-4.7)	4.2 (3.8-4.6)	4.3 (3.8-4.7)	4.2 (3.7-4.7)	0.860
**Sodium (mmol/l)**	134.0 (130.0-137.0)	134.0 (131.0-137.0)	134.0 (130.8-138.0)	134.0 (130.0-136.0)	0.398
**Platelets (Tsd/µl)**	119.0 (80.0-177.5)	86.0 (63.0-127.0)	137.0 (93.0-186.0)	120.5 (82.3-187.0)	0.003
**Leukocytes (Tsd/µl)**	7.8 (5.5-11.0)	6.1 (4.2-8.8)	8.0 (6.1-10.7)	8.1 (5.6-11.7)	0.022
**Hemoglobin (g/dl)**	10.1 (8.8-11.8)	9.3 (8.3-11.0)	10.3 (9.2-12.1)	10.3 (8.8-11.8)	0.163
**INR**	1.4 (1.3-1.7)	1.5 (1.3-1.6)	1.4 (1.3-1.7)	1.4 (1.3-1.7)	0.801
**Bilirubin (µmol/l)**	35.0 (17.0-105.0)	44.0 (20.0-109.0)	29.0 (14.0-87.0)	37.0 (17.0-112.75)	0.231
**Creatinine (µmol/l)**	120.0 (82.3-178.0)	118.0 (81.0-155.0)	138.0 (91.0-185.0)	117.5 (79.0-183.3)	0.115
**CRP (mg/l)**	19.5 (10.0-39.0)	16.0 (8.8-37.0)	20.8 (13.0-40.3)	21.0 (10.0-40.0)	0.245
**Serum-cholinesterase (kU/l)**	2.0 (1.5-2.7)	1.8 (1.4-2.7)	2.1 (1.5-2.5)	1.9 (1.4-2.7)	0.872
**Albumin (g/l)**	28.0 (24.0-32.0)	27.0 (24.5-31.5)	28.5 (25.0-33.3)	28.0 (24.0-32.0)	0.760
**BMI (kg/$m^{2}$)**	22.9 (19.8-26.8)	27.8 (24.7-33.0)	24.1 (20.6-28.4)	22.1 (19.1-25.3)	<0.001
**Diabetes mellitus**	101 (24.3)	33 (60.0)	23 (34.8)	45 (15.3)	<0.001
** Continued alcohol consumption during follow-up**	50 (12.0)	0 (0.0)	4 (6.0)	46 (15.6)	0.001
** Positive serum-ethanol**	10 (20.0)	0 (0.0)	0 (0.0)	10 (21.7)	
** Serum-ethanol (mmol/l)**	14.2 (5.1-50.0)			14.2 (5.1-50.0)	
** Positive urine-ethyl glucuronide**	8 (16.0)	0 (0.0)	1 (25.0)	7 (15.2)	
** Urine-ethyl glucuronide (mg/l)**	8.0 (2.3-68.6)		1.3 (1.3-1.3)	8.9 (5.2-71.9)	
**Medication at baseline**					
**NSBB**	178 (42.8)	24 (43.6)	28 (41.8)	126 (42.9)	0.977
**Norfloxacin**	12 (2.9)	2 (3.6)	1 (1.5)	9 (3.1)	0.738
**Rifaximin**	70 (16.8)	17 (30.9)	11 (16.4)	42 (14.3)	0.010
**Lactulose**	226 (54.3)	29 (52.7)	33 (49.3)	164 (55.8)	0.606
**Ornithine aspartate**	85 (20.4)	16 (29.1)	5 (7.5)	64 (21.8)	0.007
**Proton pump inhibitors**	321 (77.2)	42 (76.4)	52 (77.6)	227 (77.2)	0.986
**History of prior decompensation**					
**Ascites**	343 (82.1)	49 (89.1)	58 (86.6)	236 (80.3)	0.18
**Hepatic encephalopathy**	74 (17.7)	6 (10.9)	12 (17.9)	56 (19.0)	0.35
**Portal-hypertensive bleeding**	74 (17.7)	7 (12.7)	10 (14.9)	57 (19.4)	0.40

Values as n (%) or median (IQR).

* Chi-Square was used for categorical variables, ANOVA for continuous values.

ALD: Alcohol-related steatotic liver disease, BMI: Body mass index, CRP: C-reactive protein, INR: International normalized ratio, IQR: Interquartile range, MASLD: Metabolic-dysfunction associated steatotic liver disease, MELD: Model for Endstage Liver Disease, MetALD: Metabolic-dysfunction associated and alcohol-related steatotic liver disease, NSBB: Non-selective betablockers, SBP: Spontaneous bacterial peritonitis.

### Clinical events during follow-up

Concerning overall survival, 37.3% of the patients (n = 155) died or underwent LTx within one year of follow-up and a number of 148 patients was lost to follow-up within one year. Almost two thirds of patients (62.3%; n = 259) acquired at least one infection. In detail, development of SBP was observed in 39.9% (n = 166) patients. Furthermore, oHE was detected in about a third (32.0%; n = 133), 37.3% (n = 155) were readmitted and portal-hypertensive bleeding occurred in 11.5% (n = 48) of the patients within one year.

### Impact of SLD groups on mortality and cirrhosis-related complications

**Fig 2:**
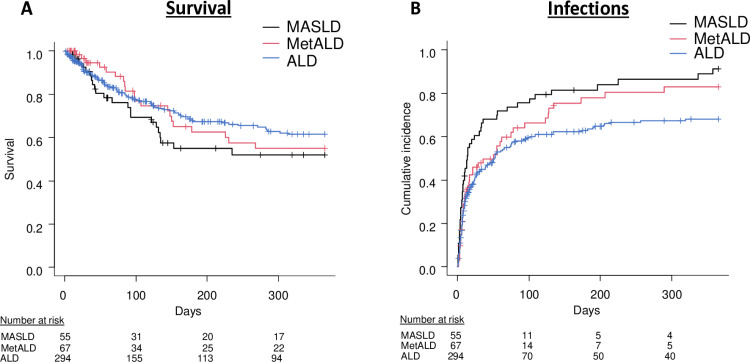
Survival and infections in the SLD groups. No differences in mortality (**A**) were detected between groups. Contrastingly, competing risk analyses demonstrated significantly higher incidences of infections in MASLD patients compared to the ALD group (**B**). ALD: Alcohol-related steatotic liver disease; MASLD: Metabolic Dysfunction-Associated steatotic liver disease; MetALD: Metabolic and alcohol-related steatotic liver disease.

Regarding the primary endpoint, neither in the 90 days-, nor in the one-year observational period, any differences in survival were observed between groups (90 days: MetALD: HR = 0.78; 95% Confidence interval (CI): 0.30–2.07; p = 0.62; ALD: HR = 0.85; 95%CI: 0.36–1.99; p = 0.71; one year: MetALD: HR = 1.03; 95%CI: 0.49–2.17; p = 0.93; ALD: HR = 0.79; 95%CI: 0.40–1.55; p = 0.49) in the multivariable analysis (Fig 2A).

Interestingly, ALD was associated with a lower risk for infections compared to MASLD in 90 days (HR = 0.60; 95%CI: 0.42–0.85; p = 0.004) and in one year (HR = 0.55; 95%CI: 0.41–0.75; p < 0.001) in the univariate competing risk analysis (Fig 2B).

In the multivariable model, the lower risk for infections in the ALD group remained statistically significant with regard to one year of follow-up (HR = 0.61; 95%CI: 0.38–0.98; p = 0.04) even after adjusting for diabetes and BMI. This was in line with the results of matched analyses (S2 Tables). Of note, incidence of infections in MetALD was higher than in ALD (68.7% vs. 56.1%; p = 0.13), but numerically lower than in MASLD (68.7% vs. 87.3%; p = 0.08). Regarding the types of infections SBP was most frequent in all three SLD groups (MASLD: n = 20 (41.7%); MetALD: n = 25 (54.3%); ALD: n = 73 (44.2%)), followed by UTI (MASLD: n = 15 (31.3%); MetALD: n = 12 (26.1%); ALD: n = 33 (20.0%) and other infections (MASLD: n = 8 (16.7%); MetALD: n = 5 (10.9); ALD: n = 28 (17.0%). Pneumonia, bloodstream infections and infections of unknown source were detected only in a minority of patients.

**Fig 3:**
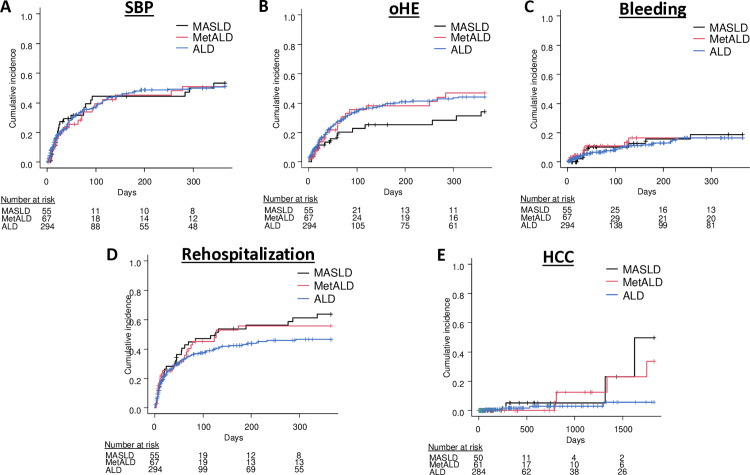
Cirrhosis-related complications. No differences were detected between the SLD groups when investigating the endpoints spontaneous bacterial peritonitis (**A**), overt hepatic encephalopathy (oHE) (**B**), portal-hypertensive bleeding (**C**), non-elective rehospitalization due to hepatic decompensation (**D**) and hepatocellular carcinoma (**E**). ALD: Alcohol-related steatotic liver disease; HCC: Hepatocellular carcinoma; MASLD: Metabolic Dysfunction-Associated steatotic liver disease; MetALD: Metabolic and alcohol-related steatotic liver disease; oHE: Overt hepatic encephalopathy; SBP: Spontaneous bacterial peritonitis.

In contrast, the risk for SBP of patients with MetALD and ALD did not differ from those with MASLD-associated cirrhosis in the multivariable competing risk model (90 days: MetALD: HR = 1.00; 95%CI: 0.51–1.97 p = 1.00; ALD: HR = 0.95; 95%CI: 0.53–1.71; p = 0.86; one year: MetALD: HR = 0.95; 95%CI: 0.52–1.73; 0.86; p = 0.86; ALD: HR = 0.86; 95%CI: 0.51–1.46; p = 0.58) (Fig 3A). Likewise, the likelihood for oHE was comparable between groups (90 days: MetALD: HR = 1.78; 95%CI: 0.75–4.24; p = 0.19; ALD: HR = 1.53; 95%CI: 0.68–3.45; p = 0.30; one year: MetALD: HR = 1.84; 95%CI: 0.86–3.95; p = 0.12; ALD: HR = 1.70; 95%CI: 0.85–3.41; p = 0.13) (Fig 3B). However, the ALD group impressed with a significantly increased risk for oHE after matching with MASLD patients (90 days: HR = 2.84; 95%CI: 1.06–7.66; p = 0.04; one year: HR = 2.52; 95%CI: 1.15–5.52; p = 0.02, S2 Tables).

Incidences of portal-hypertensive bleeding did not differ between the three SLD-groups (90 days: MetALD: HR = 1.09; 95%CI: 0.30–3.91; p = 0.90; ALD: HR = 0.53; 95%CI: 0.16–1.77; p = 0.30; one year: MetALD: HR = 0.89; 95%CI: 0.30–2.64; p = 0.83; ALD: HR = 0.60; 95%CI: 0.23–1.54; p = 0.29) (Fig 3C). Additionally, no differences in the risk for hospital readmission were detected between MetALD- and ALD-patients, compared to the MASLD group (90 days: MetALD: HR = 1.10; 95%CI: 0.54–2.22; p = 0.80; ALD: HR = 1.12; 95%CI: 0.61–2.05; p = 0.72; one year: MetALD: HR = 1.19; 95%CI: 0.64–2.22; p = 0.59; ALD: HR = 1.16; 95%CI: 0.67–2.02; p = 0.60) (Fig 3D).

Concerning HCC-development, the risk was lower in ALD-patients than in those with MASLD in the univariate analysis (MetALD: HR = 0.82; 95%CI: 0.23–2.96; p = 0.76; ALD HR = 0.21; 95%CI: 0.05–0.88; p = 0.03). However, this was not statistically significant in the multivariable approach (MetALD: HR = 0.44; 95%CI: 0.05–3.73 p = 0.45; ALD: HR = 0.17; 95%CI: 0.02–1.16; p = 0.07) (Fig 3E). Table 2 summarizes the results of the performed analyses in detail.

**Table 2:** Results of multivariable competing risk analyses. The MASLD group was treated as reference group.

	Variables	Hazard Ratio	Lower 95% CI	Upper 95% CI	p value
90 days of follow-up					
**Mortality**	**ALD**	**0.85**	**0.36**	**1.99**	**0.71**
	**MetALD**	**0.78**	**0.3**	**2.07**	**0.62**
	Age	1.02	0.99	1.05	0.18
	Sex	0.78	0.42	1.45	0.43
	MELD	1.07	1.03	1.11	<0.001
	Sodium	0.98	0.94	1.03	0.5
	Diabetes	1.5	0.8	2.79	0.2
	BMI	1.01	0.96	1.06	0.72
	S-CHE	0.38	0.25	0.58	<0.001
**Infections**	**ALD**	**0.62**	**0.37**	**1.06**	**0.08**
	**MetALD**	**0.7**	**0.39**	**1.25**	**0.22**
	Age	0.99	0.98	1.01	0.43
	Sex	0.77	0.55	1.09	1.14
	MELD	1.02	1	1.05	0.05
	Sodium	1	0.97	1.03	0.97
	Diabetes	0.94	0.64	1.4	0.77
	BMI	1	0.97	1.03	0.93
	S-CHE	0.75	0.63	0.89	0.002
	Norfloxacin	1.24	0.58	2.65	0.58
**Spontaneous bacterial peritonitis**	**ALD**	**0.95**	**0.53**	**1.71**	**0.86**
	**MetALD**	**1**	**0.51**	**1.97**	**1**
	Age	1	0.98	1.02	0.99
	Sex	0.84	0.54	1.31	0.44
	MELD	1.01	0.98	1.04	0.45
	Sodium	0.99	0.95	1.02	0.43
	Diabetes	1.31	0.84	2.04	0.23
	BMI	0.99	0.96	1.02	0.58
	S-CHE	0.66	0.51	0.86	0.002
	Norfloxacin	0.76	0.23	2.53	0.65
**Overt hepatic encephalopathy**	**ALD**	**1.53**	**0.68**	**3.45**	**0.3**
	**MetALD**	**1.78**	**0.75**	**4.24**	**0.19**
	Age	1	0.98	1.03	0.81
	Sex	0.95	0.56	1.61	0.84
	MELD	1	0.97	1.03	0.99
	Sodium	0.98	0.94	1.01	0.21
	Diabetes	1.42	0.85	2.35	0.18
	BMI	0.99	0.95	1.03	0.57
	S-CHE	0.83	0.65	1.06	0.13
	HE-prophylaxis	0.82	0.5	1.35	0.44
**Portal-hypertensive bleeding**	**ALD**	**0.53**	**0.16**	**1.77**	**0.3**
	**MetALD**	**1.09**	**0.3**	**3.91**	**0.9**
	Age	0.98	0.94	1.02	0.31
	Sex	1.73	0.59	5.02	0.32
	MELD	1.01	0.96	1.05	0.8
	Sodium	1.01	0.93	1.11	0.8
	Diabetes	0.79	0.29	2.18	0.65
	BMI	1.02	0.96	1.09	0.56
	S-CHE	0.63	0.34	1.18	0.15
	NSBB	1.62	0.68	3.83	0.27
**Rehospitalization**	**ALD**	**1.12**	**0.61**	**2.05**	**0.72**
	**MetALD**	**1.1**	**0.54**	**2.22**	**0.8**
	Age	1.01	0.99	1.03	0.55
	Sex	1	0.63	1.61	0.99
	MELD	0.96	0.93	0.99	0.01
	Sodium	0.99	0.96	1.03	0.75
	Diabetes	1.51	0.94	2.45	0.09
	BMI	1.01	0.99	1.04	0.25
	S-CHE	0.85	0.68	1.06	0.16
One year of follow-up					
**Mortality**	**ALD**	**0.79**	**0.4**	**1.55**	**0.49**
	**MetALD**	**1.03**	**0.49**	**2.17**	**0.93**
	Age	1.03	1.01	1.05	0.01
	Sex	0.96	0.59	1.55	0.86
	MELD	1.03	1	1.06	0.03
	Sodium	0.96	0.92	1	0.05
	Diabetes	1	0.61	1.66	0.99
	BMI	1.02	0.98	1.06	0.43
	S-CHE	0.48	0.36	0.64	<0.001
**Infections**	**ALD**	**0.61**	**0.38**	**0.98**	**0.04**
	**MetALD**	**0.75**	**0.45**	**1.25**	**0.27**
	Age	1	0.98	1.01	0.74
	Sex	0.8	0.58	1.09	0.16
	MELD	1.02	1	1.04	0.12
	Sodium	1	0.97	1.03	0.81
	Diabetes	1.05	0.74	1.48	0.81
	BMI	1	0.97	1.03	0.86
	S-CHE	0.83	0.72	0.96	0.01
	Norfloxacin	1.14	0.53	2.43	0.74
**Spontaneous bacterial peritonitis**	**ALD**	**0.86**	**0.51**	**1.46**	**0.58**
	**MetALD**	**0.95**	**0.52**	**1.73**	**0.86**
	Age	1.01	0.99	1.03	0.45
	Sex	1.04	0.68	1.6	0.86
	MELD	1.01	0.98	1.04	0.5
	Sodium	0.99	0.95	1.02	0.39
	Diabetes	1.27	0.85	1.91	0.25
	BMI	0.99	0.96	1.02	0.63
	S-CHE	0.75	0.61	0.93	0.01
	Norfloxacin	0.7	0.26	1.9	0.48
**Overt hepatic encephalopathy**	**ALD**	**1.7**	**0.85**	**3.41**	**0.13**
	**MetALD**	**1.84**	**0.86**	**3.95**	**0.12**
	Age	1.01	0.99	1.03	0.26
	Sex	0.87	0.55	1.36	0.53
	MELD	1.01	0.98	1.04	0.65
	Sodium	0.98	0.95	1.02	0.39
	Diabetes	1.43	0.92	2.24	0.11
	BMI	0.99	0.96	1.03	0.75
	S-CHE	0.86	0.69	1.06	0.15
	HE-	0.87	0.56	1.35	0.53
	prophylaxis				
**Portal-hypertensive bleeding**	**ALD**	**0.6**	**0.23**	**1.54**	**0.29**
	**MetALD**	**0.89**	**0.3**	**2.64**	**0.83**
	Age	0.98	0.95	1.01	0.26
	Sex	2.27	0.94	5.5	0.07
	MELD	1.01	0.98	1.04	0.5
	Sodium	1.03	0.96	1.1	0.42
	Diabetes	0.96	0.44	2.08	0.91
	BMI	1	0.95	1.06	0.88
	S-CHE	0.89	0.63	1.26	0.51
	NSBB	1.44	0.75	2.75	0.27
**Rehospitalization**	**ALD**	**1.16**	**0.67**	**2.02**	**0.6**
	**MetALD**	**1.19**	**0.64**	**2.22**	**0.59**
	Age	1.01	0.99	1.03	0.45
	Sex	1.27	0.81	1.99	0.3
	MELD	0.96	0.93	0.98	0.001
	Sodium	0.99	0.96	1.03	0.66
	Diabetes	1.37	0.88	2.13	0.16
	BMI	1.01	0.99	1.04	0.3
	S-CHE	0.91	0.76	1.09	0.32
Five years of follow-up					
**Hepatocellular carcinoma**	**ALD**	**0.17**	**0.02**	**1.16**	**0.07**
	**MetALD**	**0.44**	**0.05**	**3.73**	**0.45**
	Age	1.05	1	1.11	0.05
	Sex	1.01	0.39	2.62	0.98
	MELD	0.93	0.82	1.04	0.2
	Sodium	1.01	0.85	1.2	0.88
	Diabetes	1.27	0.23	7.03	0.79
	BMI	0.99	0.86	1.13	0.87
	S-CHE	1.14	0.6	2.16	0.68

ALD: Alcohol-related steatotic liver disease; BMI: Body mass index; CI: Confidence interval; HR: Hazard ratio; MASLD: Metabolic Dysfunction-Associated steatotic liver disease; MELD: Model for End-Stage Liver Disease; MetALD: Metabolic and alcohol-related steatotic liver disease; S-CHE: Serum-cholinesterase.

## Discussion

Since the novel SLD nomenclature has been established, only little is known about the impact of these newly defined etiologies on the clinical outcome of patients who already reached the end-stage of liver disease. To explore the clinical phenotype of the different SLD groups in advanced cirrhosis, we investigated overall survival and relevant cirrhosis-associated complications. In our large cohort of patients with decompensated SLD-related liver cirrhosis, no differences in survival were detected. However, the risk for infections seems to be increased among patients with MASLD-associated cirrhosis.

In the stage of decompensated cirrhosis, infections are relevant complications, which are linked to a potential progression of the disease course and a deteriorated prognosis [9,13]. By demonstrating a significant association between MASLD and infections, our study underscores cardiometabolic comorbidities as relevant drivers of infection vulnerability in decompensated cirrhosis. Besides the higher likelihood for infections in MASLD patients, the intermediate incidence in the MetALD group further supports the link between metabolic dysfunction and infections. For diagnosis of MASLD and MetALD, the presence of cardiometabolic criteria is required [7]. Diabetes has previously been associated with a higher likelihood for infections also in the setting of cirrhosis [14]. However, the link between MASLD and infections remained significant even in the multivariable approach including diabetes, pointing out that there are further contributing factors. Endothelial dysfunction and hemodynamic alterations, that were previously associated with the metabolic syndrome, might contribute to weakened host functions and could therefore increase risk for infections [15]. Furthermore, obesity is associated with chronic low-grade inflammation and attenuated immune functions [16,17,18]. These alterations may be attributable to the development of oxidative stress inducing pro-inflammatory adipokines from the visceral adipose tissue as well as to the linkage between obesity and elevated levels of pro-inflammatory cytokines, such as interleukin-6 (IL-6) and tumor necrosis factor alpha (TNF-alpha) [19,20,21]. Since constantly driven systemic inflammation might alter immune functions by leading to exhaustion of immune competent cells, these patients may become vulnerable for infections.

Of note, not only liver disease itself, but also the presence of diabetes and obesity has been independently linked to disruption of the intestinal microbiome, which was concomitant with elevated levels of Zonulin in a previous study, emphasizing an increased gut barrier permeability [22,23]. Besides weakening immune defense by bacterial translocation-induced systemic inflammation, the increased permeability might also act as an entry point for intestinal pathogens. Overall, the presence of cardiometabolic risk factors seems to influence the susceptibility for infections in patients with advanced liver disease. Certainly, further research is required regarding these changes in the setting of decompensated cirrhosis.

With regard to other cirrhosis specific complications, we documented an association between ALD and higher incidences of oHE in the matched analysis. These patients might be prone to develop encephalopathy due to preexisting neuronal damage caused by the neurotoxic effects of chronic alcohol misuse [24,25]. Importantly, no statistically significant differences were detected when combining the ALD and MetALD group in our analyses, suggesting that the new SLD nomenclature provides a more accurate representation of SLD-specific risk profiles. This emphasizes the relevance of implementing the novel definitions in clinical practice.

In contrast to our findings, a recent prospective observational study on patients with compensated liver disease demonstrated that the mortality and risk of liver-related complications in patients with SLD and significant fibrosis were mainly influenced by the amount of alcohol intake [26]. In detail, mortality and the risk of hepatic decompensation increased from MASLD trough MetALD to ALD and was in line with another study showing that the natural disease progression driven by cardiometabolic risk factors was lower compared to liver disease related to alcohol [27]. However, our study focuses on patients who already reached a decompensated state of cirrhosis, in which alcohol consumption may be less relevant for predicting prognosis. Furthermore, our cohort differentiates from previous studies as it only comprised a minor proportion (12.0%) of patients with active alcohol consumption. Hence, excessive ongoing alcohol intake, as present in the mentioned study [26], but not a history of alcohol abuse, as present in most ALD-patients in our cohort, might aggravate the clinical outcome compared to the other SLD groups. Given that etiological cure might have been achieved more frequently in patients with alcohol-related cirrhosis than in those with MASLD, where resolution of metabolic comorbidities remains unlikely, these patients might be predisposed to complications.

The here investigated study cohort is limited by comprising only a specific population of decompensated cirrhosis patients, who had large ascites and underwent paracentesis. Hence, our findings cannot be automatically generalized to the entire population of advanced liver diseases. Additionally, the relatively short duration of follow-up as well as the restricted sample size must be acknowledged as limitations. However, the median survival time of patients with decompensated liver cirrhosis is about two years, so that the follow-up period seems to be reasonable [28]. Furthermore, generalizability of our study is limited by a potential difference in disease severity between groups and by the predominance of ALD in the investigated cohort, whilst MASLD and MetALD were less frequent. Although the distribution of patients over the different SLD groups might reflect current real-world conditions because alcohol misuse still remains the predominant cause of liver cirrhosis in European countries [29], more balanced cohorts with higher proportions of MASLD and MetALD are required to validate our findings in further studies.

Moreover, the subdivision of patients into one out of three SLD groups remains challenging in a real-world study, as the self-reported extent of alcohol consumption might not always reflect the truth. Misclassification due to inaccurate alcohol reporting, potentially undermining the studies validity, is inherent to evaluation of the self-reported alcohol consumption. To minimize the risk for misclassification, patients with uncertain information about alcohol intake were excluded from our analyses. Moreover, alcohol consumption might vary over time, a fact that might implicate a switch between SLD groups over the lifespan of some patients. However, this is not only a limiting factor of our retrospective study, but reveals a relevant restriction of the novel SLD classification. With regard to further limitations, the possibility of missing clinical events during follow-up due to the retrospective study design has to be taken into account. To reduce the risk for missing events, patients were censored in the competing risk analyses if they were lost to follow-up.

In summary, our hypothesize-generating study suggests that the novel SLD groups exhibit partially differing clinical phenotypes in the end-stage of cirrhosis. Therefore, our results demonstrate that cardiometabolic risk factors deserve higher level attention even in patients with decompensated liver diseases. Furthermore, the influence of cardiometabolic risk factors seems to vary with the stage of liver disease.

Nevertheless, the novel SLD nomenclature not only replaces the former terminology, but introduces relevant concepts of different SLD-phenotypes, which might impact their clinical treatment at different stages of liver disease. Further studies need to evaluate whether the clinical management of SLD-patients, i.e., with regard to the risk for infections requires etiology-specific prophylactic measures.

## Supporting information

S1 Table Baseline characteristics after matching. The table displays the baseline characteristics after 1:1 propensity score matching of MASLD with ALD patients. ALD: Alcohol-related steatotic liver disease, BMI: Body mass index, CRP: C-reactive protein, INR: International normalized ratio, IQR: Interquartile range, MASLD: Metabolic-dysfunction associated steatotic liver disease, MELD: Model for End-Stage Liver Disease, MetALD: Metabolic-dysfunction associated and alcohol-related steatotic liver disease, NSBB: Non-selective betablockers, SBP: Spontaneous bacterial peritonitis.
(DOCX)

S2 Table Results of multivariable competing risk analyses after matching. MASLD and ALD patients were matched in a 1:1 ratio. ALD: Alcohol-related steatotic liver disease; BMI: Body mass index; CI: Confidence interval; HR: Hazard ratio; MASLD: Metabolic Dysfunction-Associated steatotic liver disease; MELD: Model for End-Stage Liver Disease; MetALD: Metabolic and alcohol-related steatotic liver disease; S-CHE: Serum-cholinesterase.
(DOCX)

S3 Table Competing risk analyses of combined groups. The ALD and MetALD groups were combined an compared with the MASLD patients. ALD: Alcohol-related steatotic liver disease; BMI: Body mass index; CI: Confidence interval; HR: Hazard ratio; MASLD: Metabolic Dysfunction-Associated steatotic liver disease; MELD: Model for End-Stage Liver Disease; MetALD: Metabolic and alcohol-related steatotic liver disease; S-CHE: Serum-cholinesterase.
(DOCX)

## Abbreviations:

SLD: Steatotic liver disease
MASLD: Metabolic Dysfunction-Associated steatotic liver disease
ALD: Alcohol-related steatotic liver disease
MetALD: Metabolic and alcohol-related steatotic liver disease
HR: Hazard Ratio
MELD: Model for Endstage Liver Disease
NAFLD: Nonalcoholic fatty liver disease
SBP: Spontaneous bacterial peritonitis
oHE: Overt hepatic encephalopathy
UTI: Urinary tract infection
LTx: Liver transplantation
BMI: Body mass index
S-CHE: Serum-cholinesterase
95% CI: 95% Confidence interval.
