# Supplementary material for: Differences in the incidence of cirrhosis-associated complications between MASLD, MetALD and ALD among patients with decompensated liver cirrhosis
Source: PLoS One. 2025 Jun 26;20(6):e0325673. doi: 10.1371/journal.pone.0325673 (PMC12200844; doi:10.1371/journal.pone.0325673)
Supplement: S1 Table — The table displays the baseline characteristics after 1:1 propensity score matching of MASLD with ALD patients. ALD: Alcohol-related steatotic liver disease, BMI: Body mass index, CRP: C-reactive protein, INR: International normalized ratio, IQR: Interquartile range, MASLD: Metabolic-dysfunction associated steatotic liver disease, MELD: Model for End-Stage Liver Disease, MetALD: Metabolic-dysfunction associated and alcohol-related steatotic liver disease, NSBB: Non-selective betablockers, SBP: Spontaneous bacterial peritonitis. (DOCX) [file pone.0325673.s001.docx]

**S1 Table. Baseline characteristics after matching.** The table displays the baseline characteristics after 1:1 propensity score matching of MASLD with ALD patients. ALD: Alcohol-related steatotic liver disease, BMI: Body mass index, CRP: C-reactive protein, INR: International normalized ratio, IQR: Interquartile range, MASLD: Metabolic-dysfunction associated steatotic liver disease, MELD: Model for End-Stage Liver Disease, MetALD: Metabolic-dysfunction associated and alcohol-related steatotic liver disease, NSBB: Non-selective betablockers, SBP: Spontaneous bacterial peritonitis.

|  | MASLD (n=48) | ALD (n=48) | p value* |
| --- | --- | --- | --- |
| Sex   - Male - Female | 32 (66.7)  16 (33.3) | 39 (81.3)  9 (18.8) | 0.189 |
| Age (years) | 58.1 (53.7-65.9) | 58.2 (53.0-64.6) | 0.959 |
| SBP at baseline | 6 (12.5) | 6 (12.5) | 1.000 |
| Infection at baseline | 15 (31.3) | 10 (20.8) | 0.359 |
| Hepatic encephalopathy at baseline | 5 (10.4) | 3 (6.3) | 0.687 |
|  |  |  |  |
| MELD | 29.5 (17.0-89.0) | 22.5 (14.0-29.5) | 0.001 |
| Sodium (mmol/l) | 133.5 (130.3-137.0) | 133.5 (132.0-137.0) | 0.806 |
| Platelets (Tsd/µl) | 88.0 (63.0-142.0) | 91.5 (60.3-150.0) | 0.954 |
| Leukocytes (Tsd/µl) | 6.2 (4.0-9.7) | 7.4 (4.9-10.1) | 0.335 |
| Hemoglobin (g/dl) | 9.4 (8.2-11.1) | 10.0 (9.1-11.4) | 0.216 |
| INR | 1.5 (1.4-1.7) | 1.3 (1.2-1.7) | 0.385 |
| Bilirubin (µmol/l) | 48.5 (23.0-140.5) | 26.5 (15.0-107.8) | 0.169 |
| Creatinine (µmol/l) | 119.5 (80.3-154.5) | 120.5 (70.3-168.0) | 0.601 |
| CRP (mg/l) |  |  |  |
| Serum-cholinesterase (kU/l) | 1.8 (1.4-2.7) | 2.1 (1.7-3.0) | 0.734 |
| Albumin (g/l) | 27.0 (24.0-33.0) | 29.5 (26.0-34.3) | 0.444 |
|  |  |  |  |
| BMI (kg/$\boldsymbol{m}^{\boldsymbol{2}}$) | 27.9 (24.9-33.4) | 23.3 (20.2-26.2) | <0.001 |
| Diabetes mellitus | 26 (54.2) | 27 (56.3) | 1.000 |
| Continued alcohol consumption during follow-up | 0 (0.0) | 5 (10.9) | 0.063 |
| NSBB | 17 (35.4) | 24 (50.0) | 0.210 |
| Norfloxacin | 2 (4.2) | 2 (4.2) | 1.000 |
| Rifaximin | 13 (27.1) | 6 (12.5) | 0.143 |
| Lactulose | 25 (52.1) | 28 (58.3) | 0.710 |
| Ornithine aspartate | 15 (31.9) | 12 (25.0) | 0.678 |
| Proton pump inhibitors | 35 (72.9) | 39 (81.3) | 0.454 |

Values as n (%) or median (IQR).

*McNemar test was used for categorical variables, Wilcoxon for continuous values.
