# Supplementary material for: Differences in the incidence of cirrhosis-associated complications between MASLD, MetALD and ALD among patients with decompensated liver cirrhosis
Source: PLoS One. 2025 Jun 26;20(6):e0325673. doi: 10.1371/journal.pone.0325673 (PMC12200844; doi:10.1371/journal.pone.0325673)
Supplement: S2 Table — MASLD and ALD patients were matched in a 1:1 ratio. ALD: Alcohol-related steatotic liver disease; BMI: Body mass index; CI: Confidence interval; HR: Hazard ratio; MASLD: Metabolic Dysfunction-Associated steatotic liver disease; MELD: Model for End-Stage Liver Disease; MetALD: Metabolic and alcohol-related steatotic liver disease; S-CHE: Serum-cholinesterase. (DOCX) [file pone.0325673.s002.docx]

**S2 Table. Results of multivariable competing risk analyses after matching.** MASLD and ALD patients were matched in a 1:1 ratio. ALD: Alcohol-related steatotic liver disease; BMI: Body mass index; CI: Confidence interval; HR: Hazard ratio; MASLD: Metabolic Dysfunction-Associated steatotic liver disease; MELD: Model for End-Stage Liver Disease; MetALD: Metabolic and alcohol-related steatotic liver disease; S-CHE: Serum-cholinesterase.

|  | Variables | Hazard Ratio | Lower 95% CI | Upper 95% CI | p value |
| --- | --- | --- | --- | --- | --- |
| 90 days of follow-up | | | | | |
| Mortality | **ALD**  MELD  BMI | 0.98  1.11  0.98 | 0.34  1.04  0.90 | 2.82  1.19  1.07 | 0.97  0.002  0.68 |
| Infections | **ALD**  MELD  BMI | 0.52  1.08  0.97 | 0.29  1.04  0.92 | 0.94  1.13  1.02 | 0.03  <0.001  0.25 |
| Spontaneous bacterial peritonitis | **ALD**  MELD  BMI | 0.72  1.08  0.98 | 0.35  1.02  0.93 | 1.47  1.15  1.04 | 0.36  0.01  0.58 |
| Overt hepatic encephalopathy | **ALD**  MELD  BMI | 2.84  1.02  0.98 | 1.06  0.97  0.93 | 7.66  1.08  1.03 | 0.04  0.44  0.50 |
| Portal-hypertensive bleeding | **ALD**  MELD  BMI | 1.97  1.04  1.05 | 0.40  0.93  0.99 | 9.67  1.15  1.10 | 0.40  0.52  0.11 |
| Rehospitalization | **ALD**  MELD  BMI | 1.32  0.98  1.03 | 0.66  0.94  1.00 | 2.63  1.03  1.06 | 0.43  0.42  0.03 |
|  |  |  |  |  |  |
| One year of follow-up | | | | | |
| Mortality | **ALD**  MELD  BMI | 0.98  1.10  1.03 | 0.45  1.04  0.97 | 2.14  1.16  1.08 | 0.97  <0.001  0.35 |
| Infections | **ALD**  MELD  BMI | 0.53  1.07  0.97 | 0.31  1.03  0.92 | 0.91  1.12  1.02 | 0.02  0.001  0.20 |
| Spontaneous bacterial peritonitis | **ALD**  MELD  BMI | 0.76  1.05  1.00 | 0.40  0.99  0.95 | 1.43  1.11  1.04 | 0.39  0.08  0.86 |
| Overt hepatic encephalopathy | **ALD**  MELD  BMI | 2.52  1.03  0.98 | 1.15  0.99  0.94 | 5.52  1.08  1.03 | 0.02  0.19  0.49 |
| Portal-hypertensive bleeding | **ALD**  MELD  BMI | 1.47  1.00  1.05 | 0.39  0.91  1.00 | 5.54  1.10  1.10 | 0.57  0.97  0.04 |
| Rehospitalization | **ALD**  MELD  BMI | 1.34  0.97  1.02 | 0.72  0.93  1.00 | 2.49  1.02  1.05 | 0.36  0.22  0.11 |
|  |  |  |  |  |  |
| Five years of follow-up | | | | | |
| Hepatocellular carcinoma | **ALD**  MELD  BMI | 0.43  0.75  1.13 | 0.01  0.65  0.86 | 16.87  0.86  1.48 | 0.66  <0.001  0.40 |
