# Supplementary material for: Differences in the incidence of cirrhosis-associated complications between MASLD, MetALD and ALD among patients with decompensated liver cirrhosis
Source: PLoS One. 2025 Jun 26;20(6):e0325673. doi: 10.1371/journal.pone.0325673 (PMC12200844; doi:10.1371/journal.pone.0325673)
Supplement: S3 Table — The ALD and MetALD groups were combined an compared with the MASLD patients. ALD: Alcohol-related steatotic liver disease; BMI: Body mass index; CI: Confidence interval; HR: Hazard ratio; MASLD: Metabolic Dysfunction-Associated steatotic liver disease; MELD: Model for End-Stage Liver Disease; MetALD: Metabolic and alcohol-related steatotic liver disease; S-CHE: Serum-cholinesterase. (DOCX) [file pone.0325673.s003.docx]

**S3 Table. Competing risk analyses of combined groups.** The ALD and MetALD groups were combined an compared with the MASLD patients. ALD: Alcohol-related steatotic liver disease; BMI: Body mass index; CI: Confidence interval; HR: Hazard ratio; MASLD: Metabolic Dysfunction-Associated steatotic liver disease; MELD: Model for End-Stage Liver Disease; MetALD: Metabolic and alcohol-related steatotic liver disease; S-CHE: Serum-cholinesterase.

|  | Variables | Hazard Ratio | Lower 95% CI | Upper 95% CI | p value |
| --- | --- | --- | --- | --- | --- |
| 90 days of follow-up | | | | | |
| Mortality | **ALD/MetALD**  Age  Sex  MELD  Sodium  Diabetes  BMI  S-CHE | 0.89  1.02  0.82  1.06  0.99  1.54  1.00  0.38 | 0.39  0.99  0.44  1.02  0.94  0.83  0.95  0.25 | 2.06  1.05  1.52  1.10  1.04  2.83  1.06  0.59 | 0.79  0.15  0.52  0.001  0.60  0.17  0.88  <0.001 |
| Infections | **ALD/MetALD**  Age  Sex  MELD  Sodium  Diabetes  BMI  S-CHE  Norfloxacin | 0.64  0.99  0.77  1.02  1.00  0.96  1.00  0.75  1.23 | 0.38  0.98  0.55  1.00  0.97  0.65  0.97  0.62  0.58 | 1.08  1.01  1.09  1.05  1.03  1.41  1.03  0.89  1.61 | 0.09  0.46  0.14  0.05  0.95  0.83  0.95  0.001  0.60 |
| Spontaneous bacterial peritonitis | **ALD/MetALD**  Age  Sex  MELD  Sodium  Diabetes  BMI  S-CHE  Norfloxacin | 0.96  1.00  0.84  1.01  0.99  1.32  0.99  0.66  0.75 | 0.54  0.98  0.54  0.98  0.95  0.84  0.96  0.51  0.22 | 1.71  1.02  1.30  1.04  1.02  2.06  1.02  0.86  2.51 | 0.90  0.99  0.44  0.45  0.43  0.23  0.59  0.002  0.64 |
| Overt hepatic encephalopathy | **ALD/MetALD**  Age  Sex  MELD  Sodium  Diabetes  BMI  S-CHE  HE-prophylaxis | 1.61  1.00  0.94  1.00  0.98  1.45  0.99  0.83  0.81 | 0.73  0.98  0.55  0.97  0.94  0.88  0.95  0.65  0.49 | 3.54  1.03  1.60  1.03  1.02  2.37  1.03  1.06  1.34 | 0.24  0.77  0.83  1.00  0.22  0.14  0.60  0.13  0.42 |
| Portal-hypertensive bleeding | **ALD/MetALD**  Age  Sex  MELD  Sodium  Diabetes  BMI  S-CHE  NSBB | 0.67  0.98  1.71  1.01  1.02  0.87  1.02  0.63  1.62 | 0.22  0.95  0.59  0.97  0.94  0.32  0.96  0.34  0.69 | 2.09  1.02  4.95  1.05  1.11  2.38  1.09  1.17  3.78 | 0.49  0.36  0.32  0.70  0.67  0.79  0.48  0.14  0.27 |
| Rehospitalization | **ALD/MetALD**  Age  Sex  MELD  Sodium  Diabetes  BMI  S-CHE | 1.11  1.01  1.01  0.96  0.99  1.51  1.01  0.85 | 0.62  0.99  0.63  0.93  0.96  0.94  0.99  0.68 | 1.99  1.03  1.61  0.99  1.03  2.42  1.04  1.06 | 0.72  0.55  0.98  0.01  0.75  0.09  0.26  0.16 |
|  |  |  |  |  |  |
| One year of follow-up | | | | | |
| Mortality | **ALD/MetALD**  Age  Sex  MELD  Sodium  Diabetes  BMI  S-CHE | 0.85  1.03  0.98  1.03  0.96  1.09  1.01  0.48 | 0.44  1.01  0.60  1.00  0.92  0.66  0.97  0.36 | 1.65  1.05  1.58  1.06  0.99  1.80  1.06  0.65 | 0.64  0.01  0.92  0.05  0.03  0.73  0.48  <0.001 |
| Infections | **ALD/MetALD**  Age  Sex  MELD  Sodium  Diabetes  BMI  S-CHE  Norfloxacin | 0.65  1.00  0.79  1.02  1.00  1.07  1.00  0.83  1.12 | 0.41  0.98  0.58  1.00  0.98  0.76  0.97  0.71  0.52 | 1.02  1.01  1.09  1.04  1.03  1.52  1.03  0.95  2.41 | 0.06  0.82  0.15  0.11  0.77  0.69  0.88  0.01  0.77 |
| Spontaneous bacterial peritonitis | **ALD/MetALD**  Age  Sex  MELD  Sodium  Diabetes  BMI  S-CHE  Norfloxacin | 0.89  1.01  1.04  1.01  0.99  1.28  0.99  0.75  0.69 | 0.54  0.99  0.68  0.98  0.95  0.85  0.96  0.61  0.25 | 1.47  1.03  1.59  1.04  1.02  1.93  1.02  0.93  1.88 | 0.64  0.42  0.87  0.48  0.39  0.23  0.65  0.01  0.47 |
| Overt hepatic encephalopathy | **ALD/MetALD**  Age  Sex  MELD  Sodium  Diabetes  BMI  S-CHE  HE-prophylaxis | 1.75  1.01  0.86  1.01  0.99  1.45  0.99  0.86  0.87 | 0.89  0.99  0.55  0.98  0.95  0.93  0.96  0.69  0.56 | 3.44  1.03  1.36  1.03  1.02  2.25  1.03  1.06  1.34 | 0.11  0.25  0.53  0.65  0.40  0.10  0.77  0.15  0.52 |
| Portal-hypertensive bleeding | **ALD/MetALD**  Age  Sex  MELD  Sodium  Diabetes  BMI  S-CHE  NSBB | 0.68  0.98  2.24  1.01  1.03  1.00  1.01  0.89  1.44 | 0.27  0.95  0.93  0.98  0.96  0.46  0.95  0.63  0.75 | 1.70  1.02  5.41  1.04  1.10  2.16  1.07  1.26  2.74 | 0.41  0.29  0.07  0.48  0.36  1.00  0.80  0.51  0.27 |
| Rehospitalization | **ALD/MetALD**  Age  Sex  MELD  Sodium  Diabetes  BMI  S-CHE | 1.17  1.01  1.27  0.96  0.99  1.38  1.01  0.91 | 0.69  0.99  0.81  0.93  0.96  0.90  0.99  0.76 | 1.99  1.03  1.93  0.98  1.03  2.11  1.04  1.09 | 0.57  0.45  0.30  0.001  0.67  0.14  0.29  0.32 |
|  |  |  |  |  |  |
| Five years of follow-up | | | | | |
| Hepatocellular carcinoma | **ALD/MetALD**  Age  Sex  MELD  Sodium  Diabetes  BMI  S-CHE | 0.24  1.06  0.86  0.94  1.00  1.28  1.01  1.29 | 0.04  1.01  0.31  0.86  0.85  0.69  0.89  0.69 | 1.41  1.11  2.39  1.03  1.17  2.40  1.14  2.40 | 0.11  0.03  0.78  0.22  0.96  0.43  0.90  0.43 |
